# 

**Published:** 1839-12

**Authors:** 


### TO SUBSCRIBERS.

The following list of subscribers will satisfy our professional brethren that the journal is extensively approved by the profession ; and we hope it will induce others to subscribe. If any names have been omitted of actual subscribers who have paid their subscription, it will be corrected in the eighth number when the Catalogue will be again published.

Advertisers and subscribers, who are in arrears are requested to make payment without delay, to Mr. Jahial Parmly, Treasurer, No. 11 Park Place, New York.

Our Special Patrons are hereby informed that the Publishing Committee are in need of at least one half of the amount of their subscriptions.

Ayers Daniel, Amsterdam, Mont. County, N. Y. 1
Allen Rich'd L. Sara. Springs, 1
Alcock James, N. Y. 2
Austin George, Baltimore, 1
Austin Nathan'l, Harrisonburg, Va. 2
Brewster, C. S. Paris, France. 1
Burdell John, N. Y. 20
Bridges M. K. Brooklyn, 20
Baker Elisha, N. Y. 20
Brown A. W. N. Y. 20
Bryan, Elijah, N. Y. 1
Blake Elihu, N. Y. 1
Bancroft T. L., Granville, Ohio, 1
Brown B. B. St. Louis, Mo. 2
Backus G. Nashville, Tenn. 1
Briscoe A. W., Baltimore, 1
Barstow, Wm. H., M. D., George-town, Ky. 1
Buchen David L. Hampstead, Md. 1
Bidgood Richd. W., Smith. Va. 1
Badger Felix H, Decatur, Ga. 1
Brown Solyman, N. Y. 5
Cook Doctor, Brooklyn, 1
Clute Nicholas, Louisville, Ky. 2
Chandler James, Schenec. N. Y. 1
Cobb B. C., M. D. Clarkstore, Martin's Co. N. C. 1
Cox A. L., M. D. New York, 1
Cleveland J. A., Charleston, S. C. 1
Crocker Fred'k, Sagharbour, L.I. 1
Clark, F. H., Baltimore, 1
Comegys,——Baltimore, 1
Cutler Wm. Daily, Baltimore, 1

Cutler, Sam'l Jackson, Baltimore, 1

Coleman, R. K. Baltimore, 1

Cauling & Edmunds, Balt. 1

Cobb Anson, Brooklyn, 1

Clark C., Jacksonville, Ill. 1

Clark James, Springfield, Ill. 1

Cassill John F., Upper Marion, Col. Mo, 1

Chewning F. B., Rich. Va. 2

Clark James, Lebanon, O. 1

Dexter, Doctor, Brooklyn, 1

Dunning H. H., Buffalo, 1

Davis W. H. H., Casville, Oneida Co., N. Y. 1

Duncan Archibald, N. Y. 1

Davesson Frederick A., M. D. Hillsboro, Loudon Co. Va. 1

Dodge Ezra, N. Y. 1

Dunlap Joseph, Chilicothe, O. 1

Dunbar J. R. H., M. D. Balt. 1

Desha John R., M. D. Cynthiana, Ky. 1

Esterly D., M. D. Troy, N. Y. 1

Early ——, Doctor, Lynchburg, Va. 2

Elmendorf Joseph, Pennyan, N. Y 1

Ellery E. Baltimore, 1

Evans J. N., M. D. Cynthiana, Ky. 1

Foster J. H., New-York, 2

Flagg Josiah F., M. D. Boston, 1

Frazer, Doctor, Cynthiana, Harrison Co. Ky. 1

Frink J. N. Portland, 1

Foote George, Vernon, N. Y. 1

Gilfiland, Doctor, Brooklyn, 1

Gaines Rich. W,, Charlotte C. H. Va. 1

Garrison, Doctor, Brooklyn, 1

Gallop L. F., Newport, R. I. 1

Gardette E. B., Philadel. 2

Greenwood Isaac J., New-York, 1

Green, L. T., Nashville, Tenn. 1

Goddard, W. H. Louisville, Ky. 1

Gill Bryson, Baltimore, 1

Gunnell, M. D. Washington, 1

Griffith S. & E., Louisville, Ky. 1

Geer — Rev. John A., Maryland, 1

Herd, Doctor, Brooklyn, 1

Harris Chapin A., M. D. Balt. 40

Hawes Arnold, Pawtucket, R. I. 1

Hullihen J. P. Wheeling, Va. 1

Hawes & Allen. New-York, 1

Houston P., N. Y. & Charleston, 1

Hartness Thos. L, New-York, 1

Hewlet J. W., Greensbo, N. C. 1

Holmes Oliver, Baltimore, 1

Howard F., Washington City, 1

Harris John, M. D. Georgetown, Ky. 2

Hall A. S., M. D. Scotland Neck, N. C. 1

Hubbard, E. R., Newbern, N. C. 1

Harper Sam'l, Kent Island, Md. 1

Hand G. C., Easton, Pa. 1

Humphreys Geo. W., Winchester, Va. 1

Hodgson, Doctor, Whiteplains, New-York, 1

Johnson Wm., Hagerstown, Md. 2

Jenks W. D., Frederickstown, Md. 2

King, Doctor, Brooklyn, 1

Kelly Elbridge G., Newburyport, Mass. 2

Knower Daniel, N. Y. 1

Keene Benjamin F. Hills. Ga. 1

Knapp, F H. Baltimore, 1

Latham Hiram, Brooklyn, 1

Lum John, Patterson, N. J. 1

Levett M., New York, 1

Lovejoy John, New-York, 1

Lossing B. J. New York 1

Laroque Edward, Baltimore 1

Lapham B. B., Baltimore

Lawrence J., M. D., Tarboro, N. C. 1

Leadbetter John, Alexan. D. C. 1

Lamphire Wm Alexan. D. C. 1

Marvin, Doctor, Brooklyn 1

Miller Seth P., Worcester, Mass. 1

Maynard E. — M. D. Washington City 20

Martin Chas. F., Norfolk, Va. 1

M'Kinney W. N., Fredericksburg, Va. 20

Milhau John, New York, 1

Munson W. G., New Haven, 1

Manly Horace, Cananda. N. Y. 1

Macall Leonard, M. D., Balt. 1

Miller J. H. Professor, &c. Baltimore, 1

Merryman Geo. Baltimore, 1

Manning M. E., Tarborough, N. C. 2

M'Cabe James D., Richm'd, Va. 2

McDonald G., M. D. Macon, Ga. 2

Macey Wm. M., M. D. Georgetown, Ky. 1

McAllister J. M. Albany, 1
McNaughton M. A. Albany, 1
Mason, Doctor, Brooklyn, 1
Nelson Alexander, Albany, 1
Overfield M. Winchester, Va. 2
Parker, Doctor, Brooklyn, 1
Parmly L. S., N. Orleans, 20
Parmly Jahial, New-York, 20
Parmly Jahial, Savannah, 1
Parmly Geo. W., N. Orleans, 1
Parmly David, New York, 1
Parmly Eleazar, New-Xork 40
Parmly Ludolph, Mobile, 1
Parmly Samuel, NewYork, 1
Patello Wm. H., Charlotte C. H. Va. 1
Park David N., New-York, 1
Parkhurst Wm. H., New York, 1
Peak J. M. Cooperstown, N. Y. 1
Plant Eleazar, Willimantic, Conn. 1
Pritchard P. C., Jackson, N. C. 1
Plough A. L., New Orleans, 1
Royce W. A. Poughkeepsie, N.Y. 3
Robinson E. C. Norfolk, Va. 1
Roper Lewis, M. D. Philad. 20
Rowell Chas., N. Y. 2
Roe Early, M. D. Hiilsborough, Ga. 1
Rodriguez B. A., Charles. S. C. 1
Ritter --- Washington City, 1
Robinson E. M.D. Leesburg, Ky. 1
Strickland Benj. Cleveland, O 1
Snow R. J., Buffalo, 1
Smith & Thackston, Farmville, Va. 1
Scott W. R., Raleigh, N. C. 4
Shuff P. L., M. D., Leesburg, Ky. 1
Smeds, M. D., Frankfort, Ky. 1
Stringfellow S. I. Baltimoie, 20
Taylor Edward, M, D., Bainbridge, O. 2
Tucker E. G. N. Y. 2
Trenor John, M. D. N. Y. 1
Trenor James, M. D., N. Y. 1
Tilyard H. W., Baltimore, 1
Taliaferro T. S., M. D., Maysville, Ky. 1
Taylor Joseph, Bainbridge, O. 2
Thompson — Doctor, Colum. O. 1
Thorn, Doctor, Brooklyn,
Van Praag A. S., N. Y. 1
Vincent Ezra, N. Y. 1
Willard M. T. Concord, N. H. 1
Weed J. Sacket, W. Greenfield, N. Y. 1
White Geo. H., N. Y. 1
Walker & Jones, N. Y. 20
Wanzer N. C., Auburn, N. Y. 1
Wayt John G., Richmond Va. 2
Wilson J. D. Richmond, Va. 2
Young H., Troy, N. Y. 1

---

The Publishing Committee take great pleasure in acknowledging the generous and successful efforts of Mr. M. K. Bridges, Dentist of Brooklyn, Long Island, in behalf of this Journal. Mr. Bridges has obtained the subscription of ten physicians and two other gentlemen in that sister city.

It is to be hoped that this laudable example which indicates the popularity of Mr. Bridges among his medical acquaintances, as well as his zeal for the interests of this journal and for the welfare of our profession, will be generally imitated by our brethren throughout the country, and that they will "go and do likewise." If each of our present patrons would obtain one fourth the number of subscribers which Mr. Bridges has already done, the success of this work would be placed beyond a doubt, and the Dental profession would be able to boast of a permanent magazine devoted to the interchange of useful knowledge.

### ADDRESS TO THE PROFESSION.

Of the utility of a periodical, such, as it is proposed to make this, especially to the dental profession, none can doubt. Little need, therefore, be said by way of eulogy. Its very name will, we feel assured, at once commend it to the patronage of every respectable dentist; for in none of the departments of the great medical art, is a work of this kind so much needed as in this. By reason of the scarcity of works on dentistry, many of its practitioners are unable to avail themselves of the experience of others ; and in the exercise of their profession are often compelled to depend solely on the suggestions of their own imaginations. The literature of all the other departments of medicine, although already occupying the pages of vast numbers of large volumes, is still being daily increased and enriched by periodical publications. By this means, the practitioners of medicine and surgery are kept constantly advised of all the improvements in their respective branches.

But not so with dentists, for even the works of many of the most celebrated English Authors in dental science, are either inaccessible or out of print ; and many of the best works on dentistry, being in foreign languages, are unavailable to the great majority of the profession in America. It is to supply this deficiency, and to furnish to practitioners the latest improvements in the art, that the publication of "*The American Journal of Dental Science*" has been undertaken. That the design will be appreciated by the profession, we entertain not the slightest doubt. Of the importance of constantly adding to our stock of knowledge, by keeping pace with the improvements of the art, we are all well aware. This we owe to ourselves—to the community upon whom we are dependent for support, and to the profession. Policy, and self-respect, if nothing else, require it ; and to the attainment of this object, the present publication will be found highly conducive ; for upon its pages will be spread the experience of the past, and the improvements of the present.

In making this appeal to the profession, we do it in a spirit of confidence, which, from the encouragement we have already received, we feel will not be misplaced. Many have already subscribed, some for several copies ; and all that is wanting to insure its permanent success, is, for the profession generally, throughout the United States, to extend to it a helping hand. It is, therefore, necessary, that those who intend to subscribe to it, should send on their names immediately, with the amount of subscription.

We would also, in this undertaking, respectfully solicit the aid of the medical public. To physicians residing in places where the services of the dentist cannot always be procured, the American Journal of Dental Science will be of great utility ; for, by acquainting them with the diseases of the teeth, and the means necessary for their cure, it will often enable them to treat successfully such diseases of other parts of the body as have their origin in an unhealthy condition which, otherwise, they would often be unable to accomplish. The diseases of these organs, the teeth, have, hitherto, attracted too little attention from medical men. They have not engaged as much of their attention as their importance calls for; but the want of information in medical works, on this subject, may, by the present publication, be supplied.

Nor will the usefulness of this work be altogether confined to the dental and medical practitioner : it will be found interesting and valuable to all who regard the health and preservation of their teeth as of importance. The means necessary to the attainment of this object will be carefully pointed out, so that by their proper observance, many of the diseases to which these invaluable organs are liable, may be avoided, and when the services of a dentist become necessary those skilled in the profession will be employed.

TERMS.—The AMERICAN JOURNAL OF DENTAL SCIENCE will be published monthly, on good paper, with new type, each number containing 48 octavo pages, stitched in a printed cover, making at the end of the year a volume of 576, or two volumes of 288 pages each, at $3 per annum, payable in advance. Two copies will be sent for $5.

All communications must be post paid, and directed to Solyman Brown, Dentist, 17 Park Place, New York, Secretary of the Publishing Committee.

Cash remittances should be directed to Mr. Jahial Parmly, Treasurer, No. 11 Park Place, New-York.

Editors of newspapers, to whom the first number of this Journal was sent, will, by copying the foregoing prospectus, have it continued for 12 months gratis, if they forward a copy of the paper containing the advertisement to the Secretary of the Publishing Committee.

---

Inasmuch as a sufficient number of Subscribers for single copies, or for duplicates of this Journal, did not proffer their support in time to issue a second number during the month of July, the following persons have volunteered their aid by subscribing for twenty copies or more.

If any individuals are disposed to add their names to this catalogue with a view to aid in the circulation of the Journal, they are requested to express their intention to the Publishing Committee, before the appearance of the next number.

Subscribers for less than 20 copies will not be inserted on this page.

### SPECIAL PATRONS:

| | | | |
|---|---|---|---|
| ELEAZAR PARMLY, *Dentist, N. York,* - - - | 40 | copies, | $100 |
| CHAPIN A. HARRIS, M. D. *Dentist, Baltimore* - | 40 | " | 100 |
| GEORGE KEARSING, *Goldbeater, N. York,* - - | 20 | " | 50 |
| E. NOYES, *Dentist, Baltimore,* - - - - - - - | 20 | " | 50 |
| LEVI S. PARMLY, *Dentist, New Orleans,* - - - | 20 | " | 50 |
| W. N. McKENNEY, *Dentist, Fredericksburg, Va.* - | 20 | " | 50 |
| JOHN BURDELL, *Dentist, New-York,* - - - | 20 | " | 50 |
| WALKER & JONES, *Goldbeaters, New-York,* - | 20 | " | 50 |
| SAM'L W. STOCKTON, *Dentist, Phila.* - - - | 20 | " | 50 |
| JAHIAL PARMLY, *Dentist, New-York,* - - - | 20 | " | 50 |
| E. BAKER, *Dentist, New-York,* - - - - - - - | 20 | " | 50 |
| A. WOODRUFF BROWN, *Dentist, New-York,* - | 20 | " | 50 |
| EDW'D MAYNARD, *Dentist, Washington City,* - | 20 | " | 50 |
| LEWIS ROPER, M. D. *Dentist, Philadelphia,* - | 20 | " | 50 |
| M. K. BRIDGES, *Dentist, Brooklyn,* - - - - - | 20 | " | 50 |
| S. J. STRINGFELLOW, *Dentist, Baltimore,* - - | 20 | " | 50 |
| J. A. FRAETAS, *Printer, New-York,* - - - | 20 | " | 50 |

---

### TO CONTRIBUTORS.

The Publishing Committee have received several Communications for the pages of the Journal, which, for want of room, they are compelled to reserve for the next number. Well written articles will be thankfully received from all parts of the country, especially if they discuss subjects of practical utility to the dental student.

TO THE PROFESSION.—Dentists in all parts of the United States, who desire to contribute liberally to the support of this Journal during its infancy, are respectfully informed that the fourth number will contain a list of subscribers with amount of subscription, residence, &c.; and the publishers confidently expect of such individuals that they will take several copies each, and transmit the money by mail, to Mr. Jahial Parmly, Treasurer, No. 11 Park Place, New-York. If this liberal course is not pursued by the respectable members of the profession, the publication will be unavoidably and reluctantly suspended at the termination of the present volume.

### AGENTS FOR THE AMERICAN JOURNAL OF DENTAL SCIENCE.

Doct. W. N. McKenney, *Fredericksburg, Va.*

F. B. Chewning, *Richmond, Va.*

A. Pierce, *Sussex Court House, Va.*

Chas. S. Pleasants, *Painesville, Ohio.*

David Walker & Asahel Jones, 174 *Fulton Street, New York.*

Edward Taylor, *Rochester.*

O. P. Laird, M. D., *Columbus, Ga.*

John D. Chevalier, 135 *Fulton-st. N. Y.*

Geo. Washington Parmly, *N. Orleans.*

A. W. Brown, *New-York.*

F. M. Boykin, M. D. *Smithfield, Va.*

G. McDonald, M. D. *Macon, Geo.*

E. Maynard, M. D. *Washington City.*

McAllister & McNaughton, *Albany.*

M. R. Bridges, *Brooklyn.*

Blanding & Avery, *Columbia, S. C.*

Eleazer Gidney, *England.*

S. Wheeler, M. D *Murfreesboro, N. C.*

W. H. H. Davis, M. D. *Cassville, Oneida Co. New-York.*

B. A. Rodriguez, M. D. *Charles. S. C.*

Ferguson & Goddard, *Louisville, Ky.*

